# The cornucopia of meaningful leads: Applying deep adversarial autoencoders for new molecule development in oncology

**DOI:** 10.18632/oncotarget.14073

**Published:** 2016-12-22

**Authors:** Artur Kadurin, Alexander Aliper, Andrey Kazennov, Polina Mamoshina, Quentin Vanhaelen, Kuzma Khrabrov, Alex Zhavoronkov

**Affiliations:** ^1^ Search Department, Mail.Ru Group Ltd., Moscow, Russia; ^2^ Pharmaceutical Artificial Intelligence Department, Insilico Medicine, Inc., Emerging Technology Centers, Johns Hopkins University at Eastern, Baltimore, Maryland, USA; ^3^ Big Data and Text Analysis Laboratory, Kazan Federal University, Kazan, Republic of Tatarstan, Russia; ^4^ St. Petersburg Department of V.A. Steklov Institute of Mathematics of the Russian Academy of Sciences, Petersburg, Russia; ^5^ Department of Computer Science, University of Oxford, Oxford, UK; ^6^ The Biogerontology Research Foundation, Trevissome Park, Truro TR4 8UN, UK; ^7^ Moscow Institute of Physics and Technology, Dolgoprudny, Russia

**Keywords:** generative adversarian networks, adversarial autoencoder, deep learning, drug discovery, artificial intelligence

## Abstract

Recent advances in deep learning and specifically in generative adversarial networks have demonstrated surprising results in generating new images and videos upon request even using natural language as input. In this paper we present the first application of generative adversarial autoencoders (AAE) for generating novel molecular fingerprints with a defined set of parameters. We developed a 7-layer AAE architecture with the latent middle layer serving as a discriminator. As an input and output the AAE uses a vector of binary fingerprints and concentration of the molecule. In the latent layer we also introduced a neuron responsible for growth inhibition percentage, which when negative indicates the reduction in the number of tumor cells after the treatment. To train the AAE we used the NCI-60 cell line assay data for 6252 compounds profiled on MCF-7 cell line. The output of the AAE was used to screen 72 million compounds in PubChem and select candidate molecules with potential anti-cancer properties. This approach is a proof of concept of an artificially-intelligent drug discovery engine, where AAEs are used to generate new molecular fingerprints with the desired molecular properties.

## INTRODUCTION

Despite the many advances in biomedical sciences, the productivity of research and development programs in the pharmaceutical industry is on the decline [1, 2]. The failure rates in clinical trials approach 90% for all disease categories with oncology among the categories with the lowest (5.1%) likelihood of approval (LOA) after Phase I [1, 3, 4]. One of the reasons this high failure rate is an inefficient early lead discovery process, which mostly relies on the screening of large compound libraries to identify potential leads for further preclinical development. Despite exhaustive efforts, such as modification and combination of compounds screening libraries in order to place them in relevant druggable space [5], such screening remains a blind search.

Despite many prior failures, *in silico* based approaches promise an attractive alternative to empower the industry with more efficient screening methods able to provide more reliable results at a reduced cost and timescale [6]. Although, the use of computational methods within the pharmaceutical industry is now well established, the development of new mathematical methods coupled with the availability of more powerful and cheaper computational resources, contribute to the continuous improvement and development of new techniques. Among them, Machine Learning (ML) algorithms and specifically Deep Learning (DL) methods offer a great potential for further significant advances within the industry.

In recent years DL methods demonstrated surprising results surpassing human accuracy in many tasks including image and voice recognition [7] and managed to overcome many limitations of more traditional ML approaches. From a technical point of view, modern DL techniques are structured as deep architectures, called Deep Neural Networks (DNNs). Because of this flexibility and adaptability of DNN for learning from large range of data, DNNs are now considered as an increasingly important area in the biomedical field that shows significant potential in comprehensive -omics analysis and could be useful for tackling many current issues [1, 8, 9]. Most DL-based methods require a massive amount of data for their training, optimization and validation and are often applied in most data-rich fields of biomedical sciences [10].

While the range of DL applications is diverse, two common uses are classification and prediction. One can find DL methods involving discriminative models used for classification tasks [11, 12]. These algorithms are based on the well-established backpropagation and dropout algorithms and make use of piecewise linear units [13–15] as activation functions, as they are known for having a well-behaved gradient descent.

On the other hand, there is also an increasing demand and research developed for using DL to directly generate a model that could be successfully applied, for example, to compression, denoising, inpainting, texture synthesis, semi-supervised learning, unsupervised feature learning and other tasks. However, designing deep generative models is a bigger challenge than discriminative models. This is due to the fact that initial generative models, such as restricted Boltzmann machines, Denoising autoencoders or deep Boltzmann machines [16] are probabilistic and based on a parametric specification of a probability distribution function. Training of such models requires the maximization of the log-likelihood, a function that is usually computationally intractable. As a consequence, many difficulties arise when trying to approximate the associated probabilistic computations (strategies involving the use of Markov chains (MCMC algorithm) or unrolled approximate inference networks during either training or generation of samples). Furthermore, supplementary complications may also appear when trying to leverage the benefits of piecewise linear units, the most commonly used activation function, in the generative context. In order to overcome the limitations of parametric methods, several alternative generative models have been suggested. Generative stochastic networks are an example of models that do not require the explicit representation of the likelihood while being able to generate samples from the desired distribution. As a result of the investigations performed so far, one can identify different classes of deep generative models: Deep directed graphical models, deep undirected graphical models and generative autoencoders. It is important to notice that each of these methods have advantages and disadvantages with respect to different computational and modeling steps that can be classified into five categories, that is, training, inference, sampling, likelihood evaluation and model design. As any *in silico* based approaches, these methods must be subject to validation and performance assessment. Depending on the goals, properties and specificities of the application, the global evaluation and interpretation of a generative model can be done using three independent criteria: average log-likelihood, Parzen window estimates, and visual fidelity of samples [16, 17].

The latest class of non-parametric approaches for deep generative models is known as generative adversarial network (GAN). In this new framework, initially proposed by Goodfellow et al. [18], generative models are estimated via an adversarial process. In practice, two models are simultaneously trained: a generative model G that captures the data distribution, and a discriminative model D that estimates the probability that a sample came from the training data rather than G. The training procedure for G is to maximize the probability of D making an error [19]. Thus, this framework does not correspond to the standard optimization problem as it is based on a value function that one model seeks to maximize and the other seeks to minimize. The process terminates at a saddle point that is a minimum with respect to one model's strategy and a maximum with respect to the other model's strategy [18]. Because GANs do not require an explicit representation of the likelihood, neither approximate inference nor Markov chains are necessary. Consequently, GANs provide an attractive alternative to maximum likelihood techniques.

As recently reviewed in [20, 21], the application in oncology of various types of knowledge-based *in silico* methods for predicting drug responses that require multiple kinds of -omics data for training has lead to the development and maintenance of large public databases containing curated data sets of molecular profiles of cell lines treated with the variety of small molecules. There are currently three major publicly available databases that can be used for the training of drug response prediction models. First among these is the Cancer Cell Line Encyclopedia (CCLE) [22] that contains data from more than 1000 cell lines from 36 tumor sites and drug sensitivity data from more than 11000 experiments obtained altogether from 24 anticancer drugs tested on overall 500 cell lines. Second is the Genomics of Drug Sensitivity in Cancer (GDSC) project [23], which contains data obtained from different measurements of drug sensitivity in cancer cell lines. More precisely, the GDSC contains more than 75 000 experiments that tested 138 anticancer drugs on 1000 cell lines from various cancer types. Furthermore, the GDSC also contains baseline data, that is, data obtained from untreated samples, which include gene copy number, expression data and somatic mutations in 75 genes relevant to cancer. Finally, another resource of interest is the NCI-60 cancer cell line collection [24], that provides drug screening data for thousands of drugs with potential applications in cancer therapy and 60 cell lines from nine different cancers.

In this work, we propose a deep adversarial model, specifically adversarial autoencoder, for identification and generation of new compounds that make a use of available biological and chemical data. We demonstrate that this purely insilico-based approach is capable of providing biologically relevant predictions and consequently could contribute to speed up the drug discovery process and ultimately increase the success rate within the field of anticancer therapy.

## RESULTS

**Figure 1:**
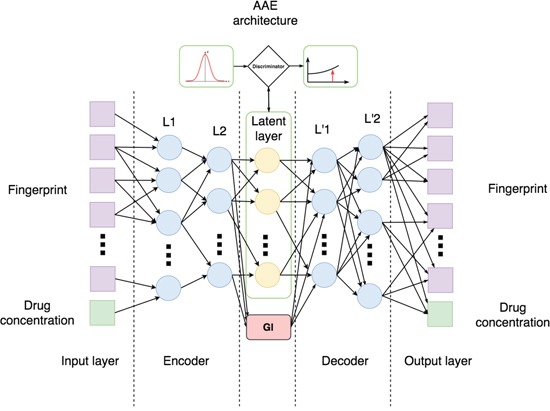
Architecture of Adversarial Autoencoder (AAE) used in this study Encoder consists of two consequent layers L1 and L2 with 128 and 64 neurons, respectively. In turn, decoder consists of layers L'1 and L'2 comprising 64 and 128 neurons. Latent layer consists of 5 neurons one of which is Growth Inhibition percentage (GI) and the other 4 are discriminated with normal distribution.

The adversarial autoencoder (AAE) architecture used in this study is depicted on Figure 1. As an input AAE uses a vector of binary fingerprints and log concentration (LCONC) of the molecule. AAE outputs concentration and a vector, consisting of probabilities assigned to each bit of the fingerprint. In the latent layer we also introduced a neuron responsible for Growth Inhibition percentage (GI), where negative values indicate the reduction in the number of tumor cells after drug treatment.

AAE was trained on fingerprint, LCONC and GI data for 6252 compounds profiled on MCF-7 cell line. After that we sampled 640 vectors from prior distribution in latent layer with 640 GI values from normal distribution N (5,1). Based on this data, we used decoder to generate 640 probability vectors with corresponding LCONC values. Then we extracted the set of probability vectors with LCONC < -5.0 M. In total, we obtained 32 vectors.

**Figure 2:**
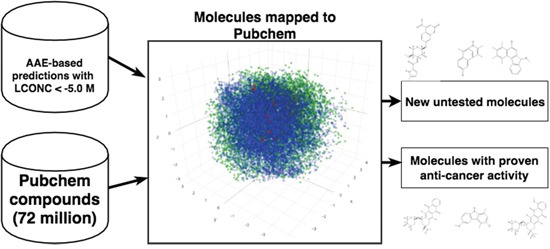
Mapping generated molecules to chemical space of Pubchem Pubchem compounds are depicted in green, training set is shown in blue and mapped predictions in red.

We screened 32 vectors them against a library of 72 million compounds derived from Pubchem [25] (Figure 2). We used the maximum likelihood function to select top 10 hits for each of the 32 vectors. This amounted for a set of 69 unique compounds (Supplementary Table 1). In order to assess the biological relevance of the our results, Pubchem BioAssay database [26] was used to identify the compounds for which anticancer activities and other relevant biomedical properties of interest have been either tested or demonstrated. This also includes several patented compounds. Although information about potential mode of action and more precisely anticancer activity is not available for all compounds, several of them are already known as anticancer agents of various kinds. Most of these compounds are related to anthracyclines (or anthracycline antibiotics). Anthracyclines are used in cancer chemotherapy to treat many cancers, including leukemias, lymphomas, stomach, uterine, ovarian, breast cancer, and lung cancers. The anthracyclines are among the most effective anticancer treatments currently available. Daunorubicinol, is an anticancer agent previously tested for treating infant with leukemia. CHEMBL519482 is another potential anticancer agent whose cytotoxicity against human KB cells has been tested using squamous cell carcinoma. Epi-daunomycin (CID:153753), also belongs to the class of anthracyclines and is classified as an antitumor antibiotic, in the treatment of neoplastic disease and blood cancers, (leukaemia and lymphoma), and many types of carcinoma and soft tissue sarcomas. Another compound, Idarubicin (CID:42890) is often traded under the denomination idamycin and idamycin PFS. This compound is classified as an antitumor antibiotic and orally administered anthracycline antineoplastic used in treatment against various types of cancers including leukemia, breast cancer and multiple myeloma. Similar to other anthracyclines, it induces histone eviction from chromatin and inhibits the activity of DNA topoisomerase II. It has been patented for numerous clinical purposes. (7R,9R)-Idarubicin (CID:151582) is another anticancer agent tested for small cell lung cancer therapies. CID:53304462 has undergone several testing phase and available bioassays demonstrate its activity for various functions including inhibitor of protein arginine methyltransferase 1 (PRMT1). Epi-daunorubicin (CID:125250) has been the subject of several studies to assess its effects against cancer such as nasopharyngeal carcinoma. CID:57620448 was patented in 2009 (patent ID US7893023) as prodrug activated by plasmin that can be used in cancer chemotherapy. CID:44398799 was tested regarding cytotoxicity properties against K562 leukemia cell line and SW620 colon cancer cell line. CID:59835410 is the subject of two patents (US2010022467and US7452901) for its activity as anticancer phosphonate analog. CID:21563452 is a synthetic compound patented (patent ID US3933827) for its ability to actively inhibit the growth of transplantable tumors and is therefore useful as cytostatic agent. CID:15573184 is also patented (patent ID US6838469) as it exhibits reduced gastrointestinal side-effects comprising a known active substance having antitumor effects. CID:59283582 is patented small molecule (patent ID US2010098691) as a composition for the treatment of cancer as several benzimidazole based anticancer agents can be used in combination with a second anti-cancer agent to obtain positive therapeutic outcomes.

Other compounds identified have been the subject of test phases and patent applications for other biomedical purposes. CID:54706490 has been tested as having antibacterial activity against ofloxacin, oxacillin, erythromycin resistant Staphylococcus aureus. Both compounds, CID:44329845 and CID:44329846, were tested *in vitro* and demonstrated antibacterial activity against Staphylococcus aureus. CID:58771432 is involved in a patent (patent ID US2006286103) for a stable antibody formulation. CID:58076509 is patented (patent ID US2012029167) as a drug using the magnetic properties of a metal salen complex. CID:15573192 is patented (patent ID US2015093398) as treatment indicates an inhibitory effect of that small molecule on the Zika virus induced caspase-3 activity that may correlate with its effect on reduction of Zika virus induced cell death. Finally, CID:57077355 has been patented as part of a method for treating migraine headaches (patent ID WO9506468).

## DISCUSSION AND PERSPECTIVES

While the use of DL methods in the biomedical field is still in its infancy and most of the applications are restricted to pure classification tasks, these techniques may transform drug discovery and biomarker development. In this work, we demonstrated how DNNs can be used not only for classification tasks but for biologically relevant generating models. The new conceptual architecture of AAE was used to develop and validate a complex DL-based work-flow capable of generating models of new compounds in cancer and oncology using drug concentrations and fingerprints as sole inputs. As a result, we predicted 69 compounds belonging to various chemical classes. The anticancer activity for our prediction have already been identified and in some cases these molecules are already used as anticancer agents for treating various cancer types including leukemia and breast cancer. This confirms the ability of this approach to provide biologically relevant results. To the best of our knowledge, this is the first application of GAN techniques within the field of cancer drug discovery. Further experimental validation is in order to assess whether the remaining predicted compounds show anticancer activity. One of the ways to evaluate the effects of the small molecule in multiple human cancers and validate the predictions could be transcriptional response analysis using signaling pathway activation analysis algorithms [27, 28] in PDX models, where human tissue is grafted into immunodeficient mice [29].

Generative capabilities of deep adversarial network techniques open the doors to new perspectives as it could contribute to overcome several limitations of current data driven computational methods. For example, we can apply GANs on transcriptomics data for the generation of new samples for a desired phenotypic groups and in chemoinformatics for the prediction of the physical, chemical, or biological properties and structures of molecules. Quantitative structure–activity relationships (QSAR) and quantitative structure–property relationships (QSPR) are still considered as the modern standard for predicting properties of novel molecules [30]. To that end, many ML-based approaches have been developed to tackle such problems, but recent results show that the DL-based methods match or outperform other state-of-the-art methods and demonstrate better predictive performance, parsimony and interpretability and web-based predictors are available on some cases [31]. Furthermore, new methods based on convolutional neural networks are able to perform predictions by directly using graphs of arbitrary size and shape as inputs rather than fixed feature vectors [32] and one can expect to see the development of more flexible deep generative architectures that can be applied directly to other structured data such as sequences, trees, graphs, and 3D structures [31, 33]. Thus, the deep adversarial network techniques could be used to improve accuracy, generative capabilities and predictive power and address several issues including computational cost, limited computation at each layer and limited information propagation across the graph [32].

Finally, target prediction and mapping of bioactive small compounds and molecules by analyzing binding affinities and chemical properties is another area of research that makes extensive use of data-driven computational methods in order to optimize the use of data available in existing repositories [34, 35]. Despite promising results and the availability of web-platforms to computationally identify new targets for uncharacterized molecules or secondary targets for known molecules such as SwissTargetPrediction [34], in general, the available methods remain too inaccurate for systematic binding predictions and physical experiments remain the state of the art for binding determination. In this field, DL-based methods, such as the recently released methods AtomNet based on deep convolutional neural networks [36] have allowed to circumvent several limitations and outperform more traditional computational methods including RFs, SVMs for QSAR and ligand-based virtual screening [37–39]. One can expect that the development of DL-methods making use of the GAN framework will also lead to significant improvement with respect to prediction accuracy and power.

## MATERIALS AND METHODS

### Data set selection

In this work, we used NCI-60 cell line assay full dose response data (released on September 2014) available at the Developmental Therapeutics Program (DTP) website of NCI/NIH (http://dtp.nci.nih.gov/index.html). The SMILES annotation for compounds generated using program CACTVS v. 3.2 was also downloaded from the DTP website. We utilised the Open Babel chemistry toolbox [40] to convert SMILES string into 166-bit Molecular ACCess System (MACCS) chemical fingerprints. In total, we generated MACCS fingerprints for a total of 6252 molecules with known growth inhibition percentage (GIPRCNT or GI) in NCI-60 assay. MACCS fingerprints were also generated from 72200431 molecules derived from Pubchem database [25].

### Design and training of the GAN

The architecture of the GAN used in this study was inspired by recent work in this field [18, 41]. According to original studies, the adversarial network and the autoencoder are trained jointly with SGD in two phases – the reconstruction phase and the regularization phase – executed on each mini-batch. In the reconstruction phase, the autoencoder updates the encoder and the decoder to minimize the reconstruction error of the inputs. In the regularization phase, the adversarial network first updates its discriminative network to tell apart the true samples (generated using the prior) from the generated samples (the hidden codes computed by the autoencoder). The adversarial network then updates its generator (which is also the encoder of the autoencoder) to confuse the discriminative network. Once the training procedure is done, the decoder of the autoencoder will define a generative model that maps the imposed prior of p(z) to the data distribution.

We divided the input layer into a fingerprint part and a concentration input neuron. So, our AAE was trained to encode and reconstruct not only molecular fingerprints, but also experimental concentrations. The Encoder consists of two consequent layers L1 and L2 with 128 and 64 neurons, respectively. The decoder consists of the two layers L'1 and L’2, comprising 64 and 128 neurons respectively. The latent layer consists of 5 neurons, one of which is the GI and the four others are discriminated with normal distribution. Since we train an encoder net to predict ‘efficiency’ against ‘cancer’ in a single neuron of latent layer, we divided the latent vector in two parts - ‘GI’ and ‘representation’. So we added a regression term to the encoder cost function. Furthermore, we restrict our encoder to map the same fingerprint to the same latent vector independently from input concentration by additional ‘manifold’ cost. Here we compute mean and variance of the concentrations through all dataset and then use them to sample concentrations for ‘manifold’ step. On each step we sample fingerprint from trainset and batch of concentration from normal distribution with given mean and variance. The training net with ‘manifold’ loss is performed by maximization of cosine similarity between ‘representations’ of similar fingerprints with different concentrations

All these changes resulted in a 5-step train iteration instead of a 3-step in AAE basic model:

a) Discriminator trained to distinguish between given latent distribution and encoded ‘representation’; b) Encoder trained to confuse Discriminator with generated ‘representations’; c) Encoder and Decoder trained jointly as Autoencoder; d) Encoder trained to fit ‘score’ part of latent vector; e) Encoder trained with ‘manifold’ cost.

The two first steps (a,b) are trained as usual adversarial networks. The Autoencoder cost function was computed as a sum of logloss [42] of fingerprint part and MSE of concentration parts and MSE was also used as a regression cost function.

The code for the AAE implemented in this paper is available at https://github.com/spoilt333/onco-aae

Authors would also like to acknowledge the many valuable biologically relevant and technical comments from reviewers during all phases of review that substantially improved the paper.

Insilico Medicine's Pharma.AI project is supported by a grant from the Life Extension Foundation 2016-LEF-AA- INSIL.

## SUPPLEMENTARY MATERIALS FIGURES AND TABLES




