# Supplementary material for: The cornucopia of meaningful leads: Applying deep adversarial autoencoders for new molecule development in oncology
Source: Oncotarget. 2016 Dec 22;8(7):10883–90. doi: 10.18632/oncotarget.14073 (PMC5355231; doi:10.18632/oncotarget.14073)
Supplement: Supplementary file 1 [file oncotarget-08-10883-s001.pdf]

The cornucopia of meaningful leads: Applying deep adversarial autoencoders for new molecule development in oncology

SUPPLEMENTARY TABLE

Supplementary Table 1: List of 69 compounds extracted by maximum likelihood with their respective Pubchem CID

See Supplementary File 1
